# Annotations

**Published:** 1895-05-04

**Authors:** 


### ANNOTATIONS.

### Private Hospitals in Dublin.

In Ireland they have an Academy of Medicine; and to the members of that Academy Dr. J. W. Moore, Professor of Medicine, has read a paper on the important and, just now, very much alive question of "Private or Home Hospitals." Dr. Moore, as becomes an Irishman, is lively and witty. He is also practical, but not very widely or deeply so. Very properly Dr. Moore objects to the conversion of private houses into hospitals, either private or otherwise; and to the expansion and elaboration of this objection his paper is largely devoted. He describes a case of conversion for us: "A house," he says, "built many years ago, in a noisy street, is rented or purchased. The exterior is touched up with a coat or two of paint, clean curtains are put in the windows, the rooms are papered and painted, the drains are inspected, perhaps relaid, the knocker is taken off, and the words 'Home Hospital' are painted on the hall door, and—hey, presto—there is our private hospital." All this may be, and no doubt is, quite true of the private hospitals of Dublin; but Dr. Moore apparently wishes us to think it is true of private hospitals generally: and that is by no means the case. Has Dr. Moore ever heard of Fitzroy House, London; a Home Hospital which, from every point of view, will bear the completest inspection, and which has a history of sixteen successful years behind it? In writing a paper for so grave and responsible a body as a Medical Academy, it would surely have been becoming, not only that some existing and well-known Home Hospitals should have been personally inspected, but that the special literature of Home Hospitals should have been studied! We congratulate Dr. Moore on his brilliancy, but we should have been better pleased if we could have congratulated him on a wider, deeper, and more practical knowledge of his subject.

### The Outcry against Pay Wards.

A letter appeared the other day in a morning newspaper, signed by A. H. Beal, who, if the Medical Directory may be trusted, is not a medical man. The letter discussed the "Pay Ward" question as it is now being developed in London hospitals, and the subject was treated with a fulness of knowledge which is unusual in a layman. If the laity, as would seem probable, are beginning to take up the question of pay wards in this fair-minded and businesslike spirit, there may yet be some hope that methods will be arrived at which will be satisfactory to all. We cannot but express our sympathy with those medical men, who, finding themselves committed to an under-paid, overcrowded, and slavishly worked profession, resent that competition for remunerative work which the gifts of the charitable enable the hospitals to enter upon. For our part, we take our stand upon two principles: first, that it is natural and justifiable for all sorts and conditions of men who are in danger of losing their lives from illness, to secure the best medical science of the times for themselves if possible; and, secondly, that all persons animated by this natural desire should pay for the best science of the times if they can. But the moment they are begun to be applied in practice our difficulties commence. No representative organisation has, as yet, really dealt with the question. The whole thing, in this country at any rate, has been left to chance. In America, Mr. Beal tells us, things are different. Here, as he insists, two or three of the hospitals have made crude attempts to establish pay systems, and have only succeeded in getting themselves into the worst possible odour throughout the whole length and breadth of the working medical profession. "Live and let live" should be the motto of hospitals, as of individuals. They ought not to take money from medical students for educating them, and then, by means of that very money, plus the gifts of the charitable, undersell them in the medical market. The subject is surely now at length ripe for representative and statesmanlike treatment. What medical organisation will take the lead in so necessary and medically popular a work?

### The Farmyard Pump.

We all know the sanitary abominations connected with the backyard pump. Even in days when water was not laid on, and was used with carefulness, the pump was far too near the cesspool, and filtration from one to the other was far too common. With the introduction of public water supplies, however, and their corollary, water-closets, the cesspools overflowed and became intolerably foul. Drains had to be laid; sewage—a compound containing disease germs of various kinds—leaked both from drains and cesspools into wells, and thus the introduction of public water supplies has almost always caused the rapid abandonment of town wells and pumps. Wells and pumps, however, still remain the common source of water supply in country places, and the common source of milk-borne typhoid in the towns to which their milk is carried. Dr. Macmartin Cameron points out in the *Sanitary Journal* how rapidly the conditions of agriculture are altering, and how important it is, in view of the large development of dairy farming, that the farmyard pump should be reformed. The amount of water used for dairy purposes is very considerable. There is a perpetual demand upon the pump, and the water in the well is so far lowered that the area from which it drains is much enlarged. And yet, while everything else is changed to meet the new demands, the position of the well remains in many instances the same, far too near the dung-heap, the pig-stye, and the conveniences, from all of which it doubtless receives a portion of its supply. Yet this water is used for washing butter, cleansing milk bowls and milk cans, and not improbably in many cases for the purpose of what one may, for the sake of euphemy, describe as "standardising" milk, that is, reducing it to such a strength as will be accepted by the sanitary authorities of the town where it is to be sold. Dr. Cameron urges, and not without good reason, that the farm pump should be reformed; that it should stand in a fenced enclosure of not less than a quarter of an acre, which should neither be manured, grazed, nor defiled in any way. Professor Koch has strongly urged the possibility of obtaining pure water from comparatively shallow wells, and Dr. Vivian Poore has shown by actual experiment that it can be done. But if this is to be so, the cesspool and the sewer must be abolished. Pumps and cesspools are, in fact, incompatible.

